# Supplementary material for: HIVprotI: an integrated web based platform for prediction and design of HIV proteins inhibitors
Source: J Cheminform. 2018 Mar 9;10:12. doi: 10.1186/s13321-018-0266-y (PMC5845081; doi:10.1186/s13321-018-0266-y)
Supplement: Supplementary file 2 — Additional file 2. Source code of HIVProtI web server. [file 13321_2018_266_MOESM2_ESM.zip › HIVprotI_Source-code/hivprotI/weka-3-6-6/doc/weka/clusterers/CheckClusterer.html]

CheckClusterer


---


|  |  |  |
| --- | --- | --- |
| **Overview** \| **Package** \| **Class** \| **Tree** \| **Deprecated** \| **Index** \| **Help** \| **Weka's home** | |  |
| **PREV CLASS**   **NEXT CLASS** | **FRAMES**    **NO FRAMES**     **All Classes** |  |
| SUMMARY: NESTED \| FIELD \| CONSTR \| METHOD | DETAIL: FIELD \| CONSTR \| METHOD |  |


---


## weka.clusterers Class CheckClusterer

```
java.lang.Object
  weka.core.Check
      weka.core.CheckScheme
          weka.clusterers.CheckClusterer
```

**All Implemented Interfaces:**: OptionHandler, RevisionHandler

---

``` public class CheckClusterer extends CheckScheme ```

Class for examining the capabilities and finding problems with
clusterers. If you implement a clusterer using the WEKA.libraries,
you should run the checks on it to ensure robustness and correct
operation. Passing all the tests of this object does not mean
bugs in the clusterer don't exist, but this will help find some
common ones.

Typical usage:

`java weka.clusterers.CheckClusterer -W clusterer_name
-- clusterer_options` 

CheckClusterer reports on the following:

- Clusterer abilities
  - Possible command line options to the clusterer
  - Whether the clusterer can predict nominal, numeric, string,
    date or relational class attributes.
  - Whether the clusterer can handle numeric predictor attributes
  - Whether the clusterer can handle nominal predictor attributes
  - Whether the clusterer can handle string predictor attributes
  - Whether the clusterer can handle date predictor attributes
  - Whether the clusterer can handle relational predictor attributes
  - Whether the clusterer can handle multi-instance data
  - Whether the clusterer can handle missing predictor values
  - Whether the clusterer can handle instance weights
- Correct functioning
  - Correct initialisation during buildClusterer (i.e. no result
    changes when buildClusterer called repeatedly)
  - Whether the clusterer alters the data pased to it
    (number of instances, instance order, instance weights, etc)
- Degenerate cases
  - building clusterer with zero training instances
  - all but one predictor attribute values missing
  - all predictor attribute values missing
  - all but one class values missing
  - all class values missing

Running CheckClusterer with the debug option set will output the
training dataset for any failed tests.

The `weka.clusterers.AbstractClustererTest` uses this
class to test all the clusterers. Any changes here, have to be
checked in that abstract test class, too.


Valid options are:

```
 -D
  Turn on debugging output.
```

```
 -S
  Silent mode - prints nothing to stdout.
```

```
 -N <num>
  The number of instances in the datasets (default 20).
```

```
 -nominal <num>
  The number of nominal attributes (default 2).
```

```
 -nominal-values <num>
  The number of values for nominal attributes (default 1).
```

```
 -numeric <num>
  The number of numeric attributes (default 1).
```

```
 -string <num>
  The number of string attributes (default 1).
```

```
 -date <num>
  The number of date attributes (default 1).
```

```
 -relational <num>
  The number of relational attributes (default 1).
```

```
 -num-instances-relational <num>
  The number of instances in relational/bag attributes (default 10).
```

```
 -words <comma-separated-list>
  The words to use in string attributes.
```

```
 -word-separators <chars>
  The word separators to use in string attributes.
```

```
 -W
  Full name of the clusterer analyzed.
  eg: weka.clusterers.SimpleKMeans
  (default weka.clusterers.SimpleKMeans)
```

```
 Options specific to clusterer weka.clusterers.SimpleKMeans:
```

```
 -N <num>
  number of clusters.
  (default 2).
```

```
 -V
  Display std. deviations for centroids.
```

```
 -M
  Replace missing values with mean/mode.
```

```
 -S <num>
  Random number seed.
  (default 10)
```

Options after -- are passed to the designated clusterer.

**Version:**
:   $Revision: 1.11 $

**Author:**
:   Len Trigg (trigg@cs.waikato.ac.nz), FracPete (fracpete at waikato dot ac dot nz)

**See Also:**: `TestInstances`

---

**Nested Class Summary**

**Nested classes/interfaces inherited from class weka.core.CheckScheme**

`CheckScheme.PostProcessor`


**Constructor Summary**

`CheckClusterer()`
  
          default constructor


| **Method Summary** | |
| --- | --- |
| `void` | `doTests()`             Begin the tests, reporting results to System.out |
| `Clusterer` | `getClusterer()`             Get the clusterer used as the clusterer |
| `java.lang.String[]` | `getOptions()`             Gets the current settings of the CheckClusterer. |
| `java.lang.String` | `getRevision()`             Returns the revision string. |
| `java.util.Enumeration` | `listOptions()`             Returns an enumeration describing the available options. |
| `static void` | `main(java.lang.String[] args)`             Test method for this class |
| `void` | `setClusterer(Clusterer newClusterer)`             Set the clusterer for testing. |
| `void` | `setOptions(java.lang.String[] options)`             Parses a given list of options. |

**Methods inherited from class weka.core.CheckScheme**

`attributeTypeToString, getNumDate, getNumInstances, getNumInstancesRelational, getNumNominal, getNumNumeric, getNumRelational, getNumString, getPostProcessor, getWords, getWordSeparators, hasClasspathProblems, setNumDate, setNumInstances, setNumInstancesRelational, setNumNominal, setNumNumeric, setNumRelational, setNumString, setPostProcessor, setWords, setWordSeparators`

**Methods inherited from class weka.core.Check**

`getDebug, getSilent, setDebug, setSilent`

**Methods inherited from class java.lang.Object**

`equals, getClass, hashCode, notify, notifyAll, toString, wait, wait, wait`

**Constructor Detail**

### CheckClusterer

```
public CheckClusterer()
```

:   default constructor


**Method Detail**

### listOptions

```
public java.util.Enumeration listOptions()
```

:   Returns an enumeration describing the available options.

    :   **Specified by:**: `listOptions` in interface `OptionHandler` **Overrides:**: `listOptions` in class `CheckScheme`
    :   **Returns:**: an enumeration of all the available options.

---


### setOptions

```
public void setOptions(java.lang.String[] options)
                throws java.lang.Exception
```

:   Parses a given list of options.


    Valid options are:

    ```
     -D
      Turn on debugging output.
    ```

    ```
     -S
      Silent mode - prints nothing to stdout.
    ```

    ```
     -N <num>
      The number of instances in the datasets (default 20).
    ```

    ```
     -nominal <num>
      The number of nominal attributes (default 2).
    ```

    ```
     -nominal-values <num>
      The number of values for nominal attributes (default 1).
    ```

    ```
     -numeric <num>
      The number of numeric attributes (default 1).
    ```

    ```
     -string <num>
      The number of string attributes (default 1).
    ```

    ```
     -date <num>
      The number of date attributes (default 1).
    ```

    ```
     -relational <num>
      The number of relational attributes (default 1).
    ```

    ```
     -num-instances-relational <num>
      The number of instances in relational/bag attributes (default 10).
    ```

    ```
     -words <comma-separated-list>
      The words to use in string attributes.
    ```

    ```
     -word-separators <chars>
      The word separators to use in string attributes.
    ```

    ```
     -W
      Full name of the clusterer analyzed.
      eg: weka.clusterers.SimpleKMeans
      (default weka.clusterers.SimpleKMeans)
    ```

    ```
     Options specific to clusterer weka.clusterers.SimpleKMeans:
    ```

    ```
     -N <num>
      number of clusters.
      (default 2).
    ```

    ```
     -V
      Display std. deviations for centroids.
    ```

    ```
     -M
      Replace missing values with mean/mode.
    ```

    ```
     -S <num>
      Random number seed.
      (default 10)
    ```

    :   **Specified by:**: `setOptions` in interface `OptionHandler` **Overrides:**: `setOptions` in class `CheckScheme`
    :   **Parameters:**: `options` - the list of options as an array of strings **Throws:**: `java.lang.Exception` - if an option is not supported

---


### getOptions

```
public java.lang.String[] getOptions()
```

:   Gets the current settings of the CheckClusterer.

    :   **Specified by:**: `getOptions` in interface `OptionHandler` **Overrides:**: `getOptions` in class `CheckScheme`
    :   **Returns:**: an array of strings suitable for passing to setOptions

---


### doTests

```
public void doTests()
```

:   Begin the tests, reporting results to System.out

    :   **Specified by:**: `doTests` in class `CheckScheme`

---


### setClusterer

```
public void setClusterer(Clusterer newClusterer)
```

:   Set the clusterer for testing.

    :   **Parameters:**: `newClusterer` - the Clusterer to use.

---


### getClusterer

```
public Clusterer getClusterer()
```

:   Get the clusterer used as the clusterer

    :   **Returns:**: the clusterer used as the clusterer

---


### getRevision

```
public java.lang.String getRevision()
```

:   Returns the revision string.

    :   **Returns:**: the revision

---


### main

```
public static void main(java.lang.String[] args)
```

:   Test method for this class

    :   **Parameters:**: `args` - the commandline options


---


|  |  |  |
| --- | --- | --- |
| **Overview** \| **Package** \| **Class** \| **Tree** \| **Deprecated** \| **Index** \| **Help** \| **Weka's home** | |  |
| **PREV CLASS**   **NEXT CLASS** | **FRAMES**    **NO FRAMES**     **All Classes** |  |
| SUMMARY: NESTED \| FIELD \| CONSTR \| METHOD | DETAIL: FIELD \| CONSTR \| METHOD |  |


---
